# Assessing sleep metrics in stroke survivors: a comparison between objective and subjective measures

**DOI:** 10.1007/s11325-024-03212-z

**Published:** 2024-12-04

**Authors:** Temmy L. T. Lo, Ian C. H. Leung, Lydia L. W. Leung, Paul P. Y. Chan, Rainbow T. H. Ho

**Affiliations:** 1 Centre on Behavioral Health, The University of Hong Kong, Pok Fu Lam, Hong Kong; 2 Belun Technology Company Limited, Sha Tin, Hong Kong; 3 Department of Social Work and Social Administration, The University of Hong Kong, Pok Fu Lam, Hong Kong

**Keywords:** Stroke, Cerebrovascular disease, Sleep disturbance, Sleep quality

## Abstract

**Introduction:**

Stroke survivors are at risk of sleep disturbance, which can be reflected in discrepancies between objective and subjective sleep measures. Given there are limited studies on this phenomenon and using portable monitoring devices is more convenient for stroke survivors to monitor their sleep, this study aimed to compare objectively measured (Belun Ring) and subjectively reported (sleep diary) sleep metrics (total sleep time (TST) and wakefulness after sleep onset (WASO)) in stroke survivors.

**Methods:**

In this cross-sectional study, thirty-five participants wore a ring-shaped pulse oximeter (Belun Ring) and kept a sleep diary for three consecutive nights in one week. The effects of various factors on TST and WASO were analyzed by linear mixed models. Systematic bias between two measures was examined by the Bland-Altman analysis.

**Results:**

TST and WASO were significantly affected by measures (*p* <.001), but not night. TST was significantly lower and WASO was significantly higher in the Belun Ring than in the sleep diary (*p* <.05). Age was the only covariate that had a significant effect on WASO (*p* <.05). The Bland-Altman analysis demonstrated positive bias in TST (29.55%; 95% *CI* [16.57%, 42.53%]) and negative bias in WASO (-117.35%; 95% *CI* [-137.65%, -97.06%]). Proportional bias was exhibited in WASO only (*r* =.31, *p* <.05).

**Conclusion:**

The findings revealed discrepancies between objective and subjective sleep measures in stroke survivors. It is recommended that objective measures be included when assessing and monitoring their sleep conditions.

## Introduction

Stroke survivors are at high risk of experiencing sleep disturbances, with an estimation of more than 50% of them exhibiting at least one type of sleep disturbance, including insomnia, sleep-related breathing disorders, and excessive daytime sleepiness [1, 2]. Individuals with sleep disorders often report discordance in objective and subjective sleep measures [3], which can also be exhibited in stroke survivors with potential sleep disturbances. Additionally, stroke survivors experience different sleep-wake cycles after stroke because of the altered neurological connection in the brain [4], and it remains unclear how neurological disorders affect the perception of sleep [3].

Given sleep disturbances are also associated with daytime fatigue, poor rehabilitation outcomes, depression, anxiety, and a higher risk of recurrent stroke in survivors [2, 5–7], it is vital to explore the differences between how stroke survivors perceive their sleep and the actual situation. Providing convenient access to assess their sleep conditions and seek medical advice when necessary is also crucial. While subjective measures can be employed to document individuals’ sleep condition, they often overestimate sleep duration [8, 9] and underestimate wake after sleep onset [10]. Therefore, there is a need for objective portable sleep monitor tools for this population to have better management of their sleep conditions.

While there is a growing body of evidence that evaluates using portable devices and home-based assessment in tracking sleep in stroke survivors [11, 12], disordered sleep of stroke survivors is still rarely treated [13]. Further evidence is needed regarding the application of portable devices with stroke survivors, and the agreement between objective and subjective measures in survivors remains unclear. This study aimed to examine the agreement in sleep metrics between objective and subjective measures in survivors and provide evidence for using portable objective sleep measures for monitoring sleep in stroke survivors.

## Methods

### Study design and participants

The study was a pilot quantitative study with a cross-sessional design with the ethics approval granted by the Human Research Ethics Committee of the University of Hong Kong (Ref. no.: EA210283). Thirty-five Chinese participants (40.0% female; age = 61.6 ± 1.23 years) with a diagnosis of a major stroke episode (48.6% ischemic stroke; 42.9% hemorrhagic stroke; duration = 92.66 ± 9.73 months) were recruited by convenience sampling from a local stroke patient self-help group from August 2021 to August 2022. They reported having sufficient cognitive and communication abilities and regular sleep for at least six hours per night, not being diagnosed with a transient ischemic attack (TIA) during their most recent stroke episode, no diagnosis of sleep disorder(s) and receiving treatment(s), no severe post-stroke disability (i.e., simplified modified Rankin Scale Questionnaire (smRSq) [14] > 4), no allergy to thermoplastic polyurethanes, and no comorbid physical issues, such as heart-related disease (e.g., arrhythmia), chronic obstructive pulmonary disease (COPD), or neuromuscular diseases (NMD). Approximately thirty participants are generally suggested for pilot studies [15].

Upon receiving written consent from participants, they responded to a questionnaire documenting their demographics (age, gender, year of stroke onset, type of stroke, and level of post-stroke disability). Participants’ demographic information and sleep metrics are listed in Table 1.

**Table 1:** Participants’ demographic and sleep metrics (*N* = 35)

Variable	M (SD)	Frequency (%)	
**Age**	61.6 (7.28)		
**Sex**			
Male		21 (60.00)	
Female		14 (40.00)	
**Type of Stroke**			
Ischemic Stroke		17 (48.60)	
Hemorrhage Stroke		15 (42.90)	
Unknown		3 (8.60)	
**Time of being Stroke (months)**	92.66 (57.56)		
**Sleep Measure Obtained from Belun Ring**	**1st Night**	**2nd Night**	**3rd Night**
	***M*** **(** ***SD*** **)**		
TIB (min)	408.05 (109.60)	411.03 (99.21)	409.90 (79.69)
TST (min)	294.20 (111.38)	281.29 (80.61)	284.95 (88.64)
SE (%)	71.02 (14.89)	69.26 (14.57)	69.32 (16.19)
WT	15.80 (5.95)	16.74 (7.58)	16.66 (6.37)
WASO (min)	114.05 (60.24)	129.74 (74.89)	124.95 (71.03)
bAHI	11.43 (5.80)	13.24 (7.62)	13.58 (7.31)
*Frequency (%)*			
Normal (AHI of < 5):	2 (6.70)	3 (9.70)	1 (3.40)
Mild OSA (AHI of 5–15):	22 (73.30)	19 (61.30)	20 (69.00)
Moderate OSA (AHI of 15–30):	6 (20.00)	7 (22.60)	7 (24.10)
Severe OSA (AHI of > 30):	0 (0.00)	2 (6.50)	1 (3.40)
ODI	11.89 (9.37)	14.79 (16.92)	10.91 (5.44)
*Frequency (%)*			
Normal (ODI of < 5):	7 (23.30)	7 (22.60)	4 (13.80)
Mild OSA (ODI of 5–15):	13 (43.30)	14 (45.20)	19 (65.50)
Moderate OSA (ODI of 15–30):	8 (26.70)	8 (25.80)	6 (20.70)
Severe OSA (ODI of > 30):	2 (6.70)	2 (6.50)	0 (0.00)
**Sleep Measure Obtained from Sleep Diary**			
TIB (min)	467.74 (109.58)	498.60 (105.78)	478.63 (97.52)
TST (min)	380.56 (131.66)	413.08 (115.80)	403.42 (135.52)
SE (%)	80.26 (22.14)	81.55 (15.35)	82.82 (22.72)
WASO (min)	46.15 (86.38)	49.96 (52.09)	35.48 (54.11)

Note. TIB, time in bed; TST, total sleep time; SE, sleep efficiency; WT, wake time; WASO, wakefulness after sleep onset; bAHI, Apnea-hypopnea Index; ODI, Oxygen Desaturation Index

### Measurements

The objective sleep metrics, including total time in bed (O-TIB), total sleep time (O-TST), sleep efficiency (O-SE), wakefulness after sleep onset (O-WASO), and respiratory event indexes (the apnea-hypopnea index; bAHI; and the oxygen desaturation index; ODI), were measured using a ring-shaped pulse oximeter– the Belun Ring and its algorithm (Belun Technology Company Limited, 2019). The ring records sleep duration and respiratory events by detecting oxygen saturation, photoplethysmography, and accelerometer signals [16, 17]. It has demonstrated satisfactory accuracy in predicting AHI and TST captured by PSG [16, 17]. Belun Ring and its AI algorithm (Belun Sleep Platform) have also been cleared by the US FDA 510(k) for obstructive sleep apnea diagnosis with sleep stages (K222579). The proprietary algorithm would generate a sleep report upon the ring being removed from at least six hours of sleep measurement and placed back on the cradle connected to the computer with the cloud-based software. Sleep data detected as poor quality or insufficiently generated suboptimal sleep reports were thus excluded from data analysis. The subjective profiles, such as sleep duration (S-TIB), sleep onset latency (S-SL), and wakefulness after sleep onset (S-WASO), were documented by a Traditional Chinese-translated sleep diary [18]. Total sleeping time (S-TST) was computed by (S-TIB)– (S-SL)– (S-WASO). Trained research assistants explained the procedures of using the Belun Ring and filling in the sleep diary. The research assistants also checked the data in both measures when participants returned their rings and diaries to them. Participants received a cash coupon of HKD100 (USD12.87) as an honorarium after completing the study.

### Statistical analysis

Out of 210 sleep reports collected (35 participants × 2 methods × 3 nights), 36 reports (17.1%) consisted of incomplete or invalid data (i.e., suboptimal report of Belun Ring). This study compared the objectively and subjectively measured TST and WASO. Three outliers, which deviated over three standard deviations (SD) from the mean, were removed. Linear mixed models were used to analyze the effects of different factors: measures (sleep diary and the Belun Ring) and nights (first, second, and third night). All factors were handled as fixed effects to examine their main effects on TST and WASO. Two-way interaction terms, measures and nights, were added to the models in a stepwise approach. Demographics of participants were included as controlled variables. The model’s intraclass correlation coefficients (ICC) were computed to estimate the within-subject correlation. The Bland-Altman analysis was employed to detect systematic bias and measure agreements, with the average of both methods ([Sleep Diary + Belun Ring]/2) versus the percentage differences between methods ([Sleep Diary - Belun Ring]/mean ×100%) [19]. Statistical significance for all tests was evaluated at a 0.05 level.

## Result

### Effects of measures and nights

Linear mixed models found that measures had significant main effects for both TST and WASO (*p* < .001), but not nights (*p* > .05). None of the interaction terms were significant (*p* > .05). For the control variables, only age (*p* < .05) had a significant main effect on WASO. Pairwise comparisons indicated higher TST in sleep diary (398.04 ± 13.95 min) than in the Belun Ring (287.20 ± 13.53 min), and lower WASO in sleep diary (36.47 ± 7.46 min) than in the Belun Ring (119.85 ± 7.22 min). The ICC for the models of TST and WASO were 0.19 and 0.25, respectively.

### Agreement between objective and subjective measures

**Fig. 1:**
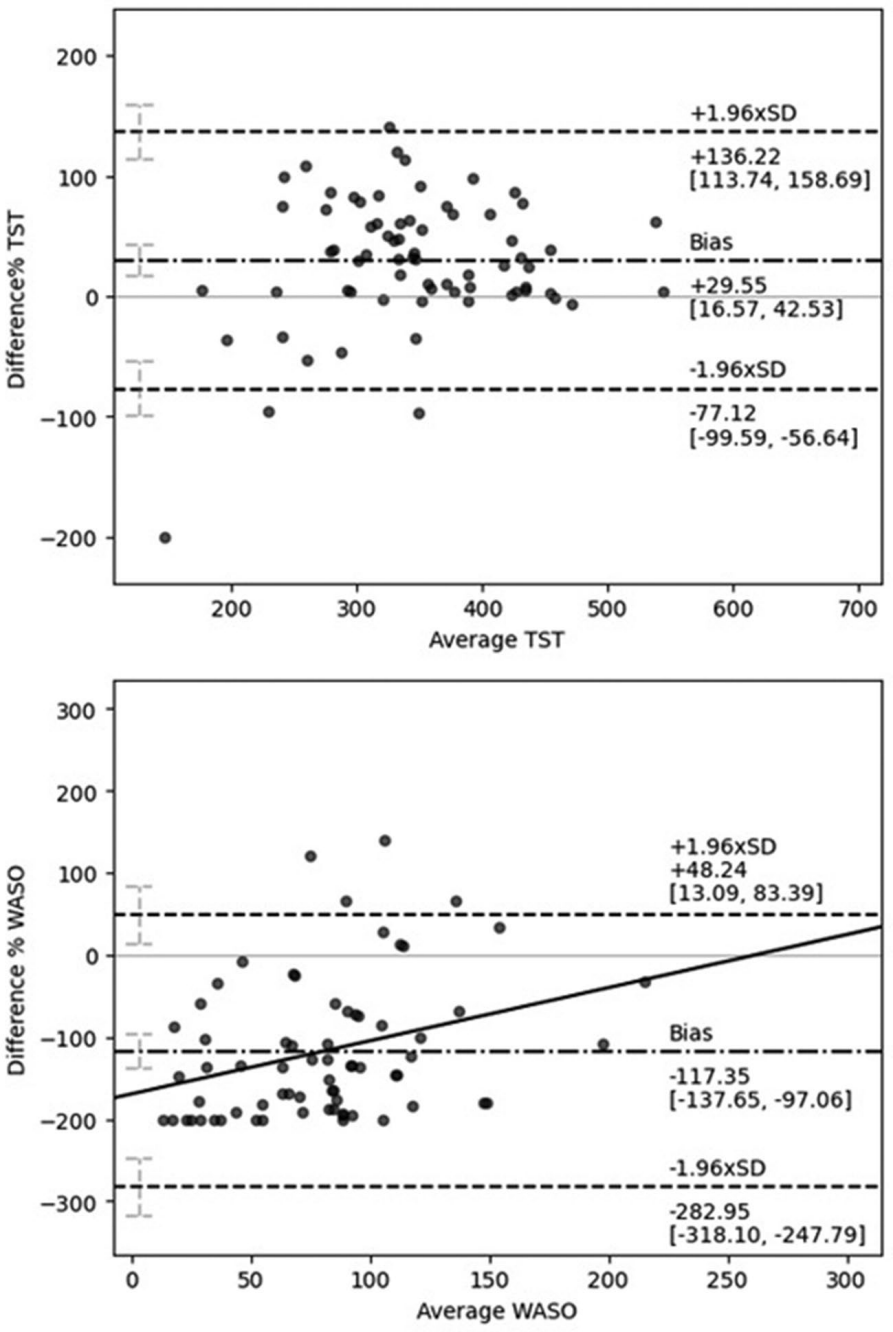
Bland-Altman Plots of TST and WASO measured by Belun Ring and Sleep Diary. *Note.* The dash-dot line represents the bias between Sleep Diary and Belun Ring. The dashed line represents LOA. 95% *CI* of bias, upper LOA, and lower LOA are indicated in brackets. The regression line is shown as the solid line if the correlation is statistically significant

The Bland-Altman analysis demonstrated a positive bias of 29.55% (95% *CI* [16.57%, 42.53%]) in TST and a negative bias of -117.35% (95% *CI* [-137.65%, -97.06%]) in WASO. Only a positive correlation was exhibited in WASO observation (*r* =.31, *p* < .05), indicating a proportional bias. The discrepancy between the two measures gradually decreased as WASO increased. Figure 1 presents the Bland-Altman plots for TST and WASO.

## Discussion

This study compared objective (Belun Ring) and subjective measures (sleep diary) in sleep metrics (TST and WASO) in stroke survivors. The preliminary findings suggested stroke survivors tended to overestimate TST and underestimate WASO, illustrating there was sleep misperception and potential sleep disturbances in stroke survivors.

Previous literature has demonstrated regular overestimations of TST [20, 21] and underestimation of WASO [22] in time-stamped sleep diaries compared to objective measures. This phenomenon can be due to retrospective bias [23] and short awakenings are difficult to recall [3]. Our findings also demonstrated a greater average underestimation in WASO than in previous studies [3]. This difference could be attributed to individual differences and the use of different devices and algorithms in processing raw sleep data. Overall, applying merely subjective measures may not fully capture the sleep conditions of stroke survivors. Using portable objective tools (such as actigraphs and ring-shaped devices), supplemented by subjective measures, can help yield a more comprehensive review of their sleep patterns.

In accordance with previous studies [1, 2], most stroke participants in this study were detected with potential sleep disturbance. Apart from identifying reporting bias in TST and WASO, the ring also detected the mean of SE was less than 85% and a majority of them (76.7–96.5%) exhibited mild to moderate sleep apnea and oxygen desaturation. The misperception of TST and WASO was also commonly found in patients with insomnia and obstructive sleep apnea [24, 25], indicating that the participants were at a high risk of experiencing sleep disturbances. Age was also a main effect of WASO, which echoed previous findings that older adults experience more fragmented sleep and difficulties in maintaining sleep [26]. Given the significant associations between sleep and rehabilitation outcomes in stroke survivors [27], sleep assessments and psychoeducation programs for cultivating sleep health should be included in stroke rehabilitation practices, particularly for older adult stroke survivors. The application of objective measures, particularly ambulatory tools, in monitoring survivors’ sleep conditions is also warranted.

## Strengths and limitations

This study is the first to apply the Belun Ring, a ring-shaped pulse oximeter, to stroke survivors and provides more evidence and alternatives to the use of ambulatory tools to measure sleep metrics in this population. This study also has several limitations. First, a comparable control group was not included in the study, preventing comparisons of sleep conditions between stroke survivors and healthy individuals. Second, the range of time after stroke onset was wide (2–221 months); stroke survivors at different stages (acute, subacute, and chronic) may have different sleep patterns and may be affected by other external factors [28]. Third, participants were recruited via convenience sampling, biasing the sample toward more motivated survivors who may be more aware of their sleep conditions. Fourth, the ring had not been validated in stroke survivors previously.

## Further implications

Further studies with a larger sample size are needed to examine the feasibility of applying ambulatory tools in monitoring stroke survivors’ sleep conditions. These tools can be applied to longitudinal studies that investigate the role of sleep in rehabilitation, such as the effect of changes in sleep architecture on rehabilitation outcomes. Further investigations can also explore the psychophysiological mechanisms of the misperception of sleep metrics. Qualitative studies can explore personal experiences of poor sleep after stroke and elucidate how sleep affects survivors’ daily routines and quality of life.

## Conclusion

This study compared objective and subjective sleep measures in stroke survivors. Poor agreements were observed between the two types of measures, particularly in TST and WASO, indicating the need to apply objective measures to obtain the actual sleep conditions of stroke survivors.

## Data Availability

The study participants did not give written consent for their data to be shared publicly. The relevant datasets are available from the corresponding author on reasonable request.

## References

[1] Cai H, Wang X-P, Yang G-Y (2021) Sleep disorders in Stroke: an update on management. Aging Disease 12:570. 10.14336/ad.2020.0707 33815883 10.14336/AD.2020.0707 PMC7990374

[2] Khot SP, Morgenstern LB (2019) Sleep and Stroke. Stroke 50:1612–1617. 10.1161/strokeaha.118.023553 31043150 10.1161/STROKEAHA.118.023553 PMC6640639

[3] Valko PO, Hunziker S, Graf K et al (2021) Sleep-wake misperception. A comprehensive analysis of a large sleep lab cohort. Sleep Med 88:96–103. 10.1016/j.sleep.2021.10.023 34742039 10.1016/j.sleep.2021.10.023

[4] Gottlieb E, Egorova N, Khlif MS et al (2020) Regional neurodegeneration correlates with sleep–wake dysfunction after stroke. Sleep 43:zsaa054. 10.1093/sleep/zsaa054 32249910 10.1093/sleep/zsaa054

[5] Ho LYW, Lai CKY, Ng SSM (2021) Contribution of sleep quality to fatigue following a stroke: a cross-sectional study. BMC Neurol 21:151. 10.1186/s12883-021-02174-z 10.1186/s12883-021-02174-z PMC8028229 33827471

[6] Fulk GD, Boyne P, Hauger M et al (2020) The impact of sleep disorders on functional recovery and participation following stroke: a systematic review and meta-analysis. Neurorehabilit Neural Repair 34:1050–1061. 10.1177/1545968320962501 10.1177/1545968320962501 33153378

[7] Liu F, Yang Y, Wang S et al (2021) Impact of sleep duration on depression and anxiety after acute ischemic stroke. Front Neurol 12:630638. 10.3389/fneur.2021.630638 33841304 10.3389/fneur.2021.630638 PMC8032928

[8] Jackson CL, Patel SR, Jackson WB et al (2018) Agreement between self-reported and objectively measured sleep duration among white, black, Hispanic, and Chinese adults in the United States: multi-ethnic study of atherosclerosis. Sleep 41:zsy057. 10.1093/sleep/zsy057 10.1093/sleep/zsy057 PMC5995218 29701831

[9] Benz F, Riemann D, Domschke K et al (2023) How many hours do you sleep? A comparison of subjective and objective sleep duration measures in a sample of insomnia patients and good sleepers. J Sleep Res 32:e13802. 10.1111/jsr.13802 36529876 10.1111/jsr.13802

[10] Lehrer HM, Yao Z, Krafty RT et al (2022) Comparing polysomnography, actigraphy, and sleep diary in the home environment: the Study of Women’s Health Across the Nation (SWAN) Sleep Study. Sleep Adv 3:zpac001. 10.1093/sleepadvances/zpac001 10.1093/sleepadvances/zpac001 PMC8918428 35296109

[11] Boulos MI, Kamra M, Colelli DR et al (2022) SLEAP SMART (Sleep Apnea Screening using Mobile Ambulatory recorders after TIA/Stroke): a randomized controlled trial. Stroke 53:710–718. 10.1161/STROKEAHA.120.033753 34628939 10.1161/STROKEAHA.120.033753

[12] Sindorf J, Szabo AL, O’Brien MK et al (2024) Wireless wearable sensors can facilitate rapid detection of sleep apnea in hospitalized stroke patients. Sleep 47:zsae123. 10.1093/sleep/zsae123 38814827 10.1093/sleep/zsae123 PMC11543614

[13] Brown DL, Jiang X, Li C et al (2019) Sleep apnea screening is uncommon after stroke. Sleep Med 59:90–93. 10.1016/j.sleep.2018.09.009 30482619 10.1016/j.sleep.2018.09.009 PMC6437010

[14] Yuan J-L, Bruno A, Li T et al (2012) Replication and extension of the simplified modified Rankin Scale in 150 Chinese stroke patients. Eur Neurol 67:206–210. 10.1159/000334849 22377778 10.1159/000334849

[15] Browne RH (1995) On the use of a pilot sample for sample size determination. Stat Med 14:1933–1940. 10.1002/sim.4780141709 8532986 10.1002/sim.4780141709

[16] Gu W, Leung L, Kwok KC et al (2020) Belun Ring platform: a novel home sleep apnea testing system for assessment of obstructive sleep apnea. J Clin Sleep Med 16:1611–1617. 10.5664/jcsm.8592 32464087 10.5664/jcsm.8592 PMC7970584

[17] Strumpf Z, Gu W, Tsai C-W et al (2023) Belun Ring (Belun Sleep System BLS-100): deep learning-facilitated wearable enables obstructive sleep apnea detection, apnea severity categorization, and sleep stage classification in patients suspected of obstructive sleep apnea. Sleep Health 9:430–440. 10.1016/j.sleh.2023.05.001 37380590 10.1016/j.sleh.2023.05.001

[18] National Heart, Lung, and Blood Institute (2019) Sleep Diary. https://www.nhlbi.nih.gov/health-topics/all-publications-and-resources/sleep-diary. Accessed 14 Feb 2024

[19] Giavarina D (2015) Understanding Bland Altman analysis. Biochemia Med 25:141–151. 10.11613/bm.2015.015 10.11613/BM.2015.015 PMC4470095 26110027

[20] Dietch JR, Taylor DJ (2021) Evaluation of the Consensus Sleep Diary in a community sample: comparison with single-channel EEG, actigraphy, and retrospective questionnaire. J Clin Sleep Med 17:1389–1399. 10.5664/jcsm.9200 33666165 10.5664/jcsm.9200 PMC8314633

[21] Takahashi N, Matsumoto T, Nakatsuka Y et al (2022) Differences between subjective and objective sleep duration according to actual sleep duration and sleep-disordered breathing: the Nagahama Study. J Clin Sleep Med 18:851–859. 10.5664/jcsm.9732 34694989 10.5664/jcsm.9732 PMC8883084

[22] Marmis R, McGoldrick-Ruth L, Kelly MR et al (2024) Comparing actigraphy and diary to measure daily and average sleep in firefighters: a Bland–Altman analysis. J Clin Sleep Med 20:497–503. 10.5664/jcsm.10916 37950454 10.5664/jcsm.10916 PMC10985296

[23] Clegg-Kraynok M, Barnovsky L, Zhou ES (2023) Real, misreported, and backfilled adherence with paper sleep diaries. Sleep Med 107:31–35. 10.1016/j.sleep.2023.04.011 37116434 10.1016/j.sleep.2023.04.011

[24] Castillo J, Goparaju B, Bianchi MT (2014) Sleep-wake misperception in sleep apnea patients undergoing diagnostic versus titration polysomnography. J Psychosom Res 76:361–367. 10.1016/j.jpsychores.2014.03.001 24745776 10.1016/j.jpsychores.2014.03.001 PMC4405154

[25] Trimmel K, Eder HG, Böck M et al (2021) The (mis)perception of sleep: factors influencing the discrepancy between self-reported and objective sleep parameters. J Clin Sleep Med 17:917–924. 10.5664/jcsm.9086 33393901 10.5664/jcsm.9086 PMC8320481

[26] Li J, Vitiello MV, Gooneratne NS (2022) Sleep in normal aging. Sleep Med Clin 17:161–171. 10.1016/j.jsmc.2022.02.007 35659071 10.1016/j.jsmc.2022.02.007

[27] Iddagoda MT, Inderjeeth CA, Chan K et al (2020) Post-stroke sleep disturbances and rehabilitation outcomes: a prospective cohort study. Intern Med J 50:208–213. 10.1111/imj.14372 31111660 10.1111/imj.14372

[28] Ferre A, Ribó M, Rodríguez-Luna D et al (2013) Strokes and their relationship with sleep and sleep disorders. Neurología 28:103–118. 10.1016/j.nrleng.2010.09.004 21163212 10.1016/j.nrl.2010.09.016

